# Impact of pH Management Interval on Biohydrogen Production from Organic Fraction of Municipal Solid Wastes by Mesophilic Thermophilic Anaerobic Codigestion

**DOI:** 10.1155/2015/590753

**Published:** 2015-12-27

**Authors:** Chaudhry Arslan, Asma Sattar, Ji Changying, Abdul Nasir, Irshad Ali Mari, Muhammad Zia Bakht

**Affiliations:** ^1^College of Engineering, Nanjing Agricultural University, Nanjing, Jiangsu 210031, China; ^2^Department of Structures and Environmental Engineering, University of Agriculture, Faisalabad 38000, Pakistan; ^3^Faculty of Agricultural Engineering, Sindh Agriculture University, Tandojam 70060, Pakistan; ^4^Institute of Food Science and Technology, University of Agriculture, Faisalabad 38000, Pakistan

## Abstract

The biohydrogen productions from the organic fraction of municipal solid wastes (OFMSW) were studied under pH management intervals of 12 h (PM12) and 24 h (PM24) for temperature of 37 ± 0.1°C and 55 ± 0.1°C. The OFMSW or food waste (FW) along with its two components, noodle waste (NW) and rice waste (RW), was codigested with sludge to estimate the potential of biohydrogen production. The biohydrogen production was higher in all reactors under PM12 as compared to PM24. The drop in pH from 7 to 5.3 was observed to be appropriate for biohydrogen production via mesophilic codigestion of noodle waste with the highest biohydrogen yield of 145.93 mL/g COD_removed_ under PM12. When the temperature was increased from 37°C to 55°C and pH management interval was reduced from 24 h to 12 h, the biohydrogen yields were also changed from 39.21 mL/g COD_removed_ to 89.67 mL/g COD_removed_, 91.77 mL/g COD_removed_ to 145.93 mL/g COD_removed_, and 15.36 mL/g COD_removed_ to 117.62 mL/g COD_removed_ for FW, NW, and RW, respectively. The drop in pH and VFA production was better controlled under PM12 as compared to PM24. Overall, PM12 was found to be an effective mean for biohydrogen production through anaerobic digestion of food waste.

## 1. Introduction

Anaerobic digestion is in practice for more than a century and specifically from the last two decades, it has been used for biological hydrogen production [1]. Although a lot of research is done in this field, still hydrogen requirement is fulfilled by other conventional processes like thermal or electrochemical, which are expensive and also not environment-friendly [2, 3]. On the other end, hydrogen produced by biological means, such as dark fermentation and photofermentation, is not enough to meet the demands, as the processes are not too efficient. Mostly, dark fermentation using mix consortia of* Clostridium* is preferred over photofermentation, which has an advantage of higher yield [4, 5]. Apart from the yield, mix consortia of* Clostridium *that can survive better under a wide range of environmental conditions are used as hydrogen producers in dark fermentation [6]. Basically,* Clostridium* is Gram-positive and spore-forming anaerobic bacteria and mix consortia of* Clostridium* are easily available in the form of sludge, which makes it a suitable economical inoculum for hydrogen production [7]. Sludge also contains hydrogen consumers, that is, methanogens, that cannot survive at higher temperature, whereas* Clostridium* can survive by forming protective spores [8]. Keeping in view the spore forming property of* Clostridium *under high temperature, heat treatment in an oven is widely opted to deactivate methanogens due to easy operation and availability [9].

Along with inoculum, feed stock is also required to produce biohydrogen by anaerobic digestion. Though a variety of feedstock are tested for biohydrogen production, like agricultural waste, municipal solid waste, and glucose, the organic fraction of municipal solid waste or food waste (FW) is getting more attraction due to higher content of volatile solids and organic matter [10–12]. The FW is abundantly available as in the year 2010, 352 Mt was produced in China, where the major contributors were restaurants and hotels as one-third of the ordered food ended in the dust bin [13, 14]. The major components of FW were rice and noodle, as 40% of FW is consisted of rice waste (RW) and noodle waste (NW) [15].

During the biohydrogen production from FW and sludge, biohydrogen production rate, bacterial growth, microbial activities, and metabolic pathways are strongly affected by pH, as the degradation of food waste occurs [16]. Volatile fatty acids (VFA) are also produced during this process, which lowers the pH of the medium, and if the pH is reduced to 4, it may cease the biohydrogen production [17, 18]. In such acidic conditions, ATP does not produce biohydrogen but maintains the neutrality among the cells [19]. On the other end, increasing the pH to specific level also increases the biohydrogen production due to an increase in bacteria growth but after that specific pH, further increase in pH may decrease ATP level, which ultimately inhibits the bacterial growth [20]. Also, the methanogen activities possibly increase at higher pH, which consumes the biohydrogen producers, and reduce the yield [21]. Initial pH of anaerobic reactor also affects the biohydrogen production process, so the initial pH of 7 is opted in most cases [11, 22, 23]. Shinya et al. [24] reported optimum pH range for biohydrogen production as 4.5 to 8.5 and further research made by Tawfik and El-Qelish [25] modified the pH range as 5 to 6.5, which was further modified to 5-6, and the findings of Zhu et al. [11] changed this range to 5.5–6. Briefly, the optimum pH conditions vary with temperature, inoculum type, feed stock, and reactor type [26].

In various studies, a specific pH value was maintained for biohydrogen production. Okamoto et al. [27] maintained pH 7 and Fang kept pH at 4.5 during whole incubation. But maintaining pH at a specific value is not an easy task, whereas an average pH value can be easily maintained by automatic pH controller [28, 29]. The specific range of pH can be maintained manually by monitoring the pH continuously with the help of pH electrode and then adjusting the pH to a desired value by using HCl or KOH [30]. Sometimes, continuous monitoring of pH is not feasible; therefore on the basis of experience and literature reviewed, many scientists maintained pH to a specific value after some interval, for example, after every twenty-four hours [18, 31]. In another approach, initial pH is maintained to such a value that, even after drop in pH, the final pH at the end of incubation remained within the optimum pH range for biohydrogen production [22, 25]. Sometimes, only maintaining initial pH is not enough, especially while working at low pH values of 5.5 or 4.5. In this situation, buffers or nutrients are added to maintain specific pH or to improve alkalinity that can slow down drop in pH [11, 32].

Along with pH, temperature is also an important environmental parameter which strongly affects the biohydrogen production. Most of the studies on biohydrogen production are conducted under mesophilic and thermophilic conditions [33]. A few studies were reported under psychrophilic conditions, where microbial electrolysis cells were developed for biohydrogen production, whereas no such a sophisticated technology is required for biohydrogen production under mesophilic as well as thermophilic temperature conditions [34].

The present study is designed in order to observe the impact of pH management interval on biohydrogen production under mesophilic thermophilic codigestion of food waste and its derivatives with sludge used as source of* Clostridium* mix consortia.

## 2. Material and Methods

### 2.1. Feedstock

Food waste was collected from Qīngzhēn dining at Engineering College, Nanjing Agricultural University, Nanjing. It was the food left on the plates after lunch/dinner consisting of rice, meat, tofu, egg, noodles bones, potato, and other vegetables. At first, bones and other foreign materials were removed followed by the separation of rice and noodles. These were ground in a meat grinder with equal proportion of water so that the resultant slurry could be used for hydrogen production [35]. The sludge was obtained from settling channel, which was sieved and washed to remove impurities and dirt [23]. Later on, the sludge was heated in an oven for 15 minutes at 100°C, in order to deactivate methanogens [36].  Table 1 contained some important aspects of feedstock.

### 2.2. Experimental Design

**Figure 1:**
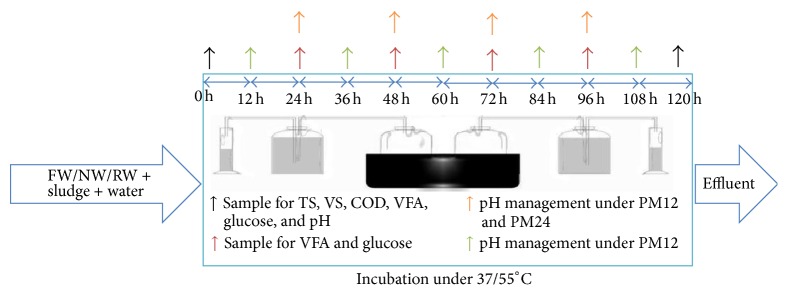
Schematic diagram for sampling and pH control.

FW, RW, and NW were mixed in equal proportion with sludge in six 550 mL digesters, two for each waste type. The working volume of each reactor was 400 mL. Initial total solids (TS) were kept to 10% and pH was initially maintained at 7 for each reactor. These reactors were placed in two water baths: one at mesophilic temperature (37 ± 0.1°C) and the other at thermophilic temperature (55 ± 0.1°C). Two sets of experiments (in duplicate) were performed [11, 34]. In the first set of experiments, pH was maintained to 7 after every 24 hours, and in second set of experiments, pH was maintained to 7 after every 12 hours. For ease of representation, 12-hour pH management was coded as PM12 and 24-hour pH management as PM24. The schematic diagram for sampling and pH control is shown in Figure 1.

### 2.3. Analytic Methods and Kinetic Modeling

The biohydrogen production volume was measured by connecting each reactor with a measuring bottle containing 3% NaOH solution that could remove other gases and water vapors. The volume of NaOH displaced out was measured by measuring cylinder as a volume of biohydrogen produced in mL [37–39]. The total solids (TS), volatile solids (VS), chemical oxygen demand (COD), and volatile fatty acids (VFA) were measured according to standard methods [40]. The phenol sulfuric acid method was used to measure glucose content [41]. For TS, VS, and COD, samples were taken before and after incubation, whereas for VFA and glucose, samples were taken with glass syringe after every twenty-four hours [42]. For pH monitoring and control, 5 mL sample was taken and the pH was monitored with pH meter. The pH was adjusted to 7 by adding 3 M NaOH or 3 M HCl with a syringe [30].

### 2.4. Assay Methods

The kinetic modeling was done by a modified Gompertz equation that was used for cumulative biohydrogen measurement [43]

(1) $$\begin{array}{c} H=P\;exp\left\{\;-\exp\left[\;\frac{R_{m}e}{P}\left(\;\lambda -t\;\right)+1\;\right]\;\right\}, \end{array}$$

where *H*, *t*, *P*, *R*_*m*_, *λ*, and *e* represent cumulative hydrogen production (mL), incubation time (h), hydrogen production potential, maximum hydrogen production rate (mL/h), lag phase duration (h), and 2.71828, respectively. The equation was solved by using Matlab (ver. 2010 a).

## 3. Results and Discussion

### 3.1. Biohydrogen Production

**Figure 2:**
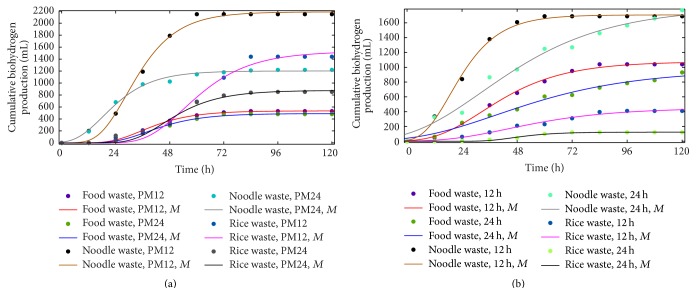
Biohydrogen production under 12 h and 24 h management interval. (a) 37°C; (b) 55°C. *M* modeled curve on the basis of modified Gompertz equation.

**Figure 3:**
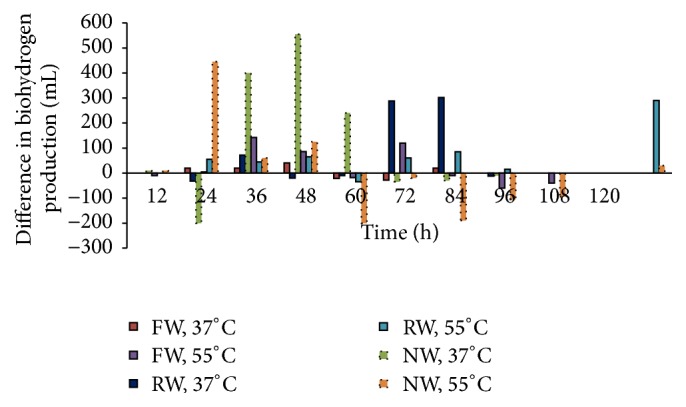
Comparison of biohydrogen production under PM12 over PM24.

The biohydrogen production was modeled by using modified Gompertz equation and the results obtained were drawn in comparison with experimental results. It is clear form Figure 2 that the start of biohydrogen production was independent of pH management interval as both were under the same conditions during the first 12 hours of incubation. On the other end, the effect of increasing temperature varies as feedstock changes. The increase in temperature from 37°C to 55°C caused an early start of biohydrogen production in FW reactor whereas the same increase in temperature did not affect the initiation of biohydrogen production in case of NW rectors. The biohydrogen production in RW reactors was also not affected by the increase in temperature, but the production started after 12 hours of incubation as compared to NW where it started during 12 hours of incubation. The biohydrogen production was ceased in most of the reactors after 72 hours of incubation, which is in agreement with the previous studies [32, 34]. The impact of pH management interval on biohydrogen production can be observed in Figure 3, where the differences in biohydrogen production under both pH managements were drawn on 12-hourly basis. In case of FW reactor, at 37°C, PM12 was less dominant over PM24 till 48 hours of incubation, but after this time, PM24 remained dominant till 72 hours of incubation. Increasing the temperature from 37°C to 55°C changed the domination from 48 hours to 72 hours, but as a whole, PM12 remained dominant. When feedstock was changed from FW to NW, the change in domination was observed after 60 hours of incubation at 37°C with one exception that PM24 was dominated between 12 and 24 hours of incubation. On the other end, increase in temperature from 37°C to 55°C for NW reactor dominated the PM24 after 48 hours of incubation and as a whole there was little difference observed in both pH managements. The rice waste represented different situation where the PM12 was highly dominated between 60 and 84 hours of incubation at 37°C temperature conditions. When the temperature was increased to 55°C, PM12 remained dominated except between 48 and 60 hours where PM24 dominated.

The kinetic parameters assessed on the basis of the modified Gompertz equation are listed in Table 2. For FW, the highest value of *R*_*m*_ was 19.76 mL/h observed at 55°C, PM12, and produced cumulative biohydrogen of 1076 mL. In case of NW, the mesophilic value of *R*_*m*_ was 65.66 mL/h at PM12, which is much higher than 43.08 mL/h observed at PM24. The increase in pH management duration and temperature form 37°C to 55°C decreased the value of *R*_*m*_ for RW that can be observed in Table 2. On the whole, an increase in pH management time decreased the cumulative biohydrogen production for all waste types. The increase in temperature from 37°C to 55°C decreased *R*_*m*_ for NW and RW, which ultimately reduced the biohydrogen production [18]. An increase in temperature increased the biohydrogen production for FW, although the thermophilic *R*_*m*_ under PM24 was smaller than the mesophilic *R*_*m*_ under PM24. It was due to lag phase duration that was small under thermophilic condition as compared to mesophilic conditions and also due to longer production time that can be observed in Figure 2 and Table 2.

### 3.2. Drop in pH

**Figure 4:**
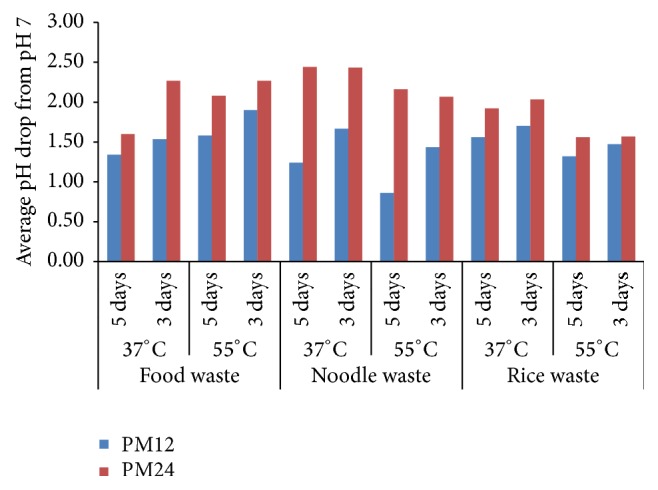
Drop in pH during PM12 and PM24.

The production of biohydrogen occurred in acidification phase, which decreased the pH of the reactor [26], as most of the hydrogen production was observed during 72 hours of incubation after which the drop in pH was reduced (Figure 4). The average drop in pH during 5 days was lower than that observed during first 3 days, meaning thereby that the intensity of drop in pH reduced during last two days of incubation. The reduction in biohydrogen production and decrease in intensity of drop in pH indicated the possibility of activation of methanogenic bacteria, which were deactivated initially by heat treatment [44, 45]. It is obvious that the drop in pH increases when pH management interval increases; still the drop in pH difference for PM24 was not so higher than PM12 specifically during 72 hours of incubation. As compared to other reactors, NW rector has higher average pH drop during 5 days under PM12 and PM24. It was because of reduction in drop of pH, especially after 72 hours of incubation under PM12 that ultimately caused higher difference between the drop of pH under PM12 and PM24. The drop in pH decreased with an increase in temperature for RW and NW. In the reactors, which attained higher biohydrogen production such as thermophilic FW, mesophilic NW, and mesophilic RW, average drop in pH was from 7 to 5.1, 5.3, and 5.3, respectively [25].

### 3.3. Biohydrogen Yield

Biohydrogen yield was calculated by dividing the cumulative biohydrogen production with COD_removed_ and the highest biohydrogen yield of 145.93 mL/g COD_removed_ was obtained by NW reactor at 37°C under PM12. The biohydrogen yields of all tested wastes in the present study are listed in Table 2, which are in agreement with the previous studies, although the pH management method used was different [25, 46]. On the other end, Wongthanate and Chinnacotpong [47] obtained biohydrogen yield of 44.83 mL/g COD under no pH control conditions which was 89.67 mL/g COD obtained in the present study from FW under PM12, indicating the positive impact of pH management.

The increase in pH management interval decreased the biohydrogen yield for FW which was higher under mesophilic conditions as compared to thermophilic conditions, that is, 60% and 6.42% decrease in biohydrogen yield at 37°C and 55°C, respectively. At the same time, increase in pH management interval also increased the average drop in pH from 5.1 to 4.7 under thermophilic conditions, whereas the drop in biohydrogen yield due to such pH shift was not so high under the same temperature conditions. So the optimum pH range of biohydrogen production from FW under thermophilic conditions could be considered as 7 to 4.7. On the other end, the increase in temperature from 37°C to 55°C increased biohydrogen yield by 66.67% and 142% under PM12 and PM24, respectively [30].

For NW, changing from PM12 to PM24 caused a 57.61% decrease in biohydrogen yield under mesophilic conditions. But the situation was different under thermophilic conditions where 10.36% increase in biohydrogen yield was observed by changing from PM12 to PM24. During biohydrogen production at 55°C, the average drop in pH from 7 was 5.6 and 4.9 under PM12 and PM24, respectively. As the thermophilic biohydrogen yield was higher than the mesophilic biohydrogen yield under PM24, so the optimum pH range for biohydrogen production from NW was observed between 7 and 4.9.

In case of RW, the biohydrogen yield decreased from PM12 to PM24, that is, 61.67% and 135.48% decrease in biohydrogen yield at 37°C and 55°C, respectively. Similarly, an increase in temperature from 37°C to 55°C decreased biohydrogen yield by 62.46% and 74.23% under PM12 and PM24, respectively [18]. The highest experimental biohydrogen yield of 117.61 mL/g COD_removed_ from RW was observed under mesophilic conditions with PM12. Keeping in view the average pH drop, optimum pH range of biohydrogen production from RW was observed between 7 and 5.3.

### 3.4. Glucose Consumption

**Figure 5:**
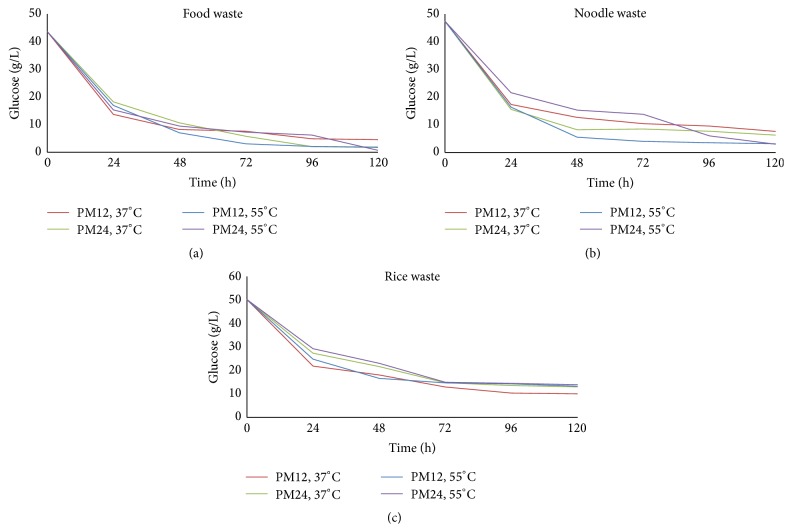
Glucose consumption during incubation.

The mix consortia of* Clostridium* metabolize glucose into pyruvate that is further oxidized to ferredoxin, which is ultimately converted to hydrogen and volatile fatty acids [48, 49]. Due to such metabolism, there was a sudden drop in glucose concentration with little biohydrogen production during the first 24 h of incubation in all reactors that was also observed in previous studies [32, 50]. As it is already discussed that most of the biohydrogen was produced during 72 h of incubation and during the same interval, as a whole, average glucose consumption was 80% (Figure 5) that is in agreement with the previous studies [32, 39].

The increase in temperature from mesophilic to thermophilic and increase in pH management interval increased the glucose consumption for FW and NW as shown in Figures 5(a) and 5(b). Keeping in view the above fact, FW and NW thermophilic reactors under PM24 have higher glucose consumption as compared to other reactors as biohydrogen production was not fully ceased in these reactors till the end of incubation (Figure 2(b)). As the gas production was continued in these reactors, so the glucose consumption also remained higher as compared to other reactors, especially after 72 hours of incubation (Figures 5(a) and 5(b)). On the other end, RW reactors represented opposite trends as observed for FW and NW with respect to temperature and pH management (Figure 5(c)). The highest consumption of glucose for RW was observed in mesophilic reactor under PM12 having higher biohydrogen production potential as compared to other RW reactors.

### 3.5. VFA Production

**Figure 6:**
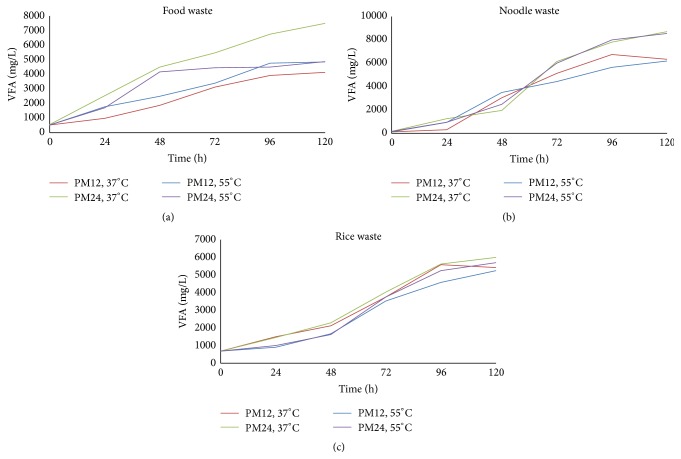
VFA production during incubation.

The VFA production represented an increasing trend with incubation time as observed in studies made by other researchers [18, 22]. The overall increase in VFA was also observed when pH management was changed from PM12 to PM24 as shown in Figure 6. The mesophilic FW and NW reactors under PM24 represented a higher VFA concentration at the end of incubation as compared to other reactors. That might be associated with the conversion of glucose into VFA instead of biohydrogen, especially after 60 hours of incubation (Figure 6(a) and 6(b)). On the other end, an increase in temperature from 37°C to 55°C increased the VFA for FW under PM12, but it decreased the VFA for all other reactors (Figure 6). Such a variable impact of temperature on VFA is due to variation in test conditions like different feedstock and pH environment and so forth, as observed in previous studies [30, 51].

There was a sudden increase in VFA concentration between 24 h and 48 h of incubation in FW reactor under PM24, which considerably reduced the biohydrogen production as compared to the FW reactor under PM12 (Figures 2 and 6(a)), whereas such increase in VFA was observed in the NW and RW reactors between 48 h and 72 h of incubation under PM24 but did not considerably affect biohydrogen production in RW reactors, as a similar increase in VFA concentration was also observed under PM12 (Figure 6(c)).

After 72 h of incubation, the increase in VFA under PM24 was much higher than PM12 in all reactors under mesophilic as well as thermophilic conditions. The NW reactors have the highest concentration of VFA as observed previously [39]. Overall, it was observed that PM12 not only controls the production of VFA but also increases the biohydrogen production potential of waste types tested in present study.

## 4. Conclusion

The effect of pH management interval on biohydrogen production from the organic fraction of municipal solid waste was studied under mesophilic and thermophilic conditions. Managing the pH after specific intervals was found to be a practical approach to enhancing biohydrogen production. The biohydrogen yields of 145.93 mL/g COD_removed_, 89.67 mL/g COD_removed_, and 117.61 mL/g COD_removed_ were obtained for FW, NW, and RW, respectively, under PM12 which were higher than those obtained under PM24. Increasing in temperature from 37°C to 55°C was observed to be an effective mean to enhance the biohydrogen yield for FW only. PM12 was more effective than PM24 to control the production of VFA. The results obtained in this study are useful for designing pH operating conditions for anaerobic reactor in order to produce biohydrogen.

## Figures and Tables

**Table 1:** Properties of test materials.

Parameter	Unit	Sludge	Food waste	Rice waste	Noodles waste
TS	%	58.59	30.32	39.88	31.54
VS	%	2.87	26.9	39.30	28.51
Glucose	g/L	2.49	65.77	79.65	63.73
COD	g/L	50	147.5	105	132
Total alkalinity	mg/L	3700	550	500	450
VFA (mg/L)	mg/L	13950	2475	9000	1500
pH	—	7.1	4.5	5.3	4.3

**Table 2:** Kinetic parameters and biohydrogen yield.

Waste type	Temperature	pH management interval	*P*	*R*_*m*_	*λ*	*R* ^2^	Biohydrogen yield	SHPR
	(°C)	(h)	(mL)	(mL/h)	(h)		mL/g COD_removed_	mL/g VS_removed_/h
Food waste	37	12	534.9	15.36	22.34	0.9998	56.31	1.90
		24	490.1	13.34	24.1	0.9969	39.20	1.56
	55	12	1076	19.79	12.45	0.9971	89.67	1.95
		24	981.2	10.09	3.22	0.9885	85.32	0.96

Noodle waste	37	12	2189	65.66	16.33	0.9935	145.93	3.47
		24	1193	43.08	9.32	0.991	91.77	2.31
	55	12	1712	48.94	4.63	0.994	122.28	2.14
		24	1723	25.56	8.56	0.982	132.54	1.28

Rice waste	37	12	1529	34.49	37.73	0.9867	117.62	2.35
		24	876	23.62	31.31	0.9819	73	1.97
	55	12	448.20	6.44	18.02	0.9779	44.82	0.67
		24	122.90	4.25	35.02	0.9897	15.36	0.53

SHPR stands for specific biohydrogen production rate.
